# Recent Advances in the Physicochemical Properties and Biotechnological Application of *Vitreoscilla* Hemoglobin

**DOI:** 10.3390/microorganisms9071455

**Published:** 2021-07-07

**Authors:** Fei Yu, Xinrui Zhao, Ziwei Wang, Luyao Liu, Lingfeng Yi, Jingwen Zhou, Jianghua Li, Jian Chen, Guocheng Du

**Affiliations:** 1 Key Laboratory of Industrial Biotechnology, Ministry of Education, School of Biotechnology, Jiangnan University, 1800 Lihu Road, Wuxi 214122, China; 7190201070@stu.jiangnan.edu.cn (F.Y.); 1022180104@stu.jiangnan.edu.cn (Z.W.); 1024180215@stu.jiangnan.edu.cn (L.L.); 1023180116@stu.jiangnan.edu.cn (L.Y.); zhoujw1982@jiangnan.edu.cn (J.Z.); lijianghua@jiangnan.edu.cn (J.L.); jchen@jiangnan.edu.cn (J.C.); 2 Science Center for Future Foods, Jiangnan University, 1800 Lihu Road, Wuxi 214122, China; 3 Key Laboratory of Carbohydrate Chemistry and Biotechnology, Ministry of Education, Jiangnan University, 1800 Lihu Road, Wuxi 214122, China

**Keywords:** *Vitreoscilla* hemoglobin, high-cell-density fermentation, physicochemical properties, metabolic regulation, expressional tactics, applications

## Abstract

*Vitreoscilla* hemoglobin (VHb), the first discovered bacterial hemoglobin, is a soluble heme-binding protein with a faster rate of oxygen dissociation. Since it can enhance cell growth, product synthesis and stress tolerance, VHb has been widely applied in the field of metabolic engineering for microorganisms, plants, and animals. Especially under oxygen-limited conditions, VHb can interact with terminal oxidase to deliver enough oxygen to achieve high-cell-density fermentation. In recent years, with the development of bioinformatics and synthetic biology, several novel physicochemical properties and metabolic regulatory effects of VHb have been discovered and numerous strategies have been utilized to enhance the expression level of VHb in various hosts, which greatly promotes its applications in biotechnology. Thus, in this review, the new information regarding structure, function and expressional tactics for VHb is summarized to understand its latest applications and pave a new way for the future improvement of biosynthesis for other products.

## 1. Introduction

*Vitreoscilla* hemoglobin (VHb) is the first bacterial hemoglobin discovered in gram-negative bacterium *Vitreoscilla* sp. C1 [1]. *Vitreoscilla* was found in oxygen-limited conditions like stagnant ponds and decaying vegetable matter [2,3], but it is strictly aerobic based on the special VHb to adapt to hypoxic conditions. VHb was originally named “cytochrome *o* (Cyo)” because of some similar properties with cytochromes [4,5]. Subsequently, the amino acid sequencing of “Cyo” was completed and showed that it had a high homology with eukaryotic hemoglobins [2].

VHb is a single-domain hemoglobin (SDHb) that is different from the two other two kinds of bacterial hemoglobins, FHbs (flavohemoglobins, a VHb-like globin fused with flavin-binding domain) and trHbs (truncated hemoglobins, a single-domain hemoglobin approximately 20% smaller than SDHb) [6]. Based on the unique structure of VHb, it can efficiently bind and transport oxygen to the respiratory chain by interacting with terminal oxidase, especially under oxygen-limited conditions [6]. In addition, VHb also can interact with transcriptional regulators responsible for oxygen response, triggering oxidative phosphorylation in the cells [6].

Based on its powerful oxygen transport capacity, VHb has been widely applied in the field of metabolic engineering for microorganisms, plants and animals. By enhancing the regeneration of ATP and NAD^+^ and improving the activity of the TCA cycle [7,8], VHb can be used to promote the growth of microbial, plant and animal cells [7,9], improve the synthesis of target products under oxygen-limited conditions [10], and increase the effect of microorganisms on bioremediation [11].

In recent years, with the development of bioinformatics and synthetic biology, several novel physicochemical properties and functions of VHb were discovered and numerous strategies were utilized to enhance the expression level of VHb in various hosts, resulting in its wide application in biotechnology. Therefore, in this review, the information of structure and functions for VHb are summarized to make VHb become a promising and practical tool in metabolic engineering.

## 2. Biochemical Function of VHb

### 2.1. The Oxygen-Binding Property of VHb

VHb is a homodimer composed of two identical subunits (146 amino acids for each subunit) and two molecules of *b*-type heme. In the early studies, VHb was considered as a cytochrome *o* [1]. Subsequently, researchers gradually recognized that VHb is a kind of hemoglobin based on its primary structure, spectral properties, and oxygen binding kinetics [2]. Under different environmental conditions, VHb can present in three different states: oxidized state, reduced state, and oxygenated state. When the iron atom in the heme of VHb is in the ferrous state, it presents in a reduced state and can reversibly combine with the oxygen. The oxygenated state is the transition state between the reduced state and oxidized state and is also the most important stable state that participates in oxygen related metabolic pathways and improves the efficiency of oxidative phosphorylation in the respiratory cells [12,13].

Compared with other eukaryotic hemoglobins, the rate constant of VHb binding to oxygen (*k_on_* = 78 μM^−1^s^−1^) is in the average level, but the dissociation rate constant of VHb and oxygen (*k_off_* = 5000 s^−1^) is hundreds of times higher, meaning VHb is apt to release a large amount of oxygen [13]. In *Vitreoscilla* and *Escherichia coli*, the cellular VHb localizes in the periplasmic space and close to cell membrane, which facilitates it functioning as a respirator to transport oxygen to the cell membrane under hypoxic conditions [14]. Whereas in yeast, it was confirmed by two-hybrid experiments that VHb can interact with subunit I of cytochrome *bo* ubiquinol oxidase and enhance its activity [15].

### 2.2. The Activity of Terminal Oxidase and Peroxidase

On the one hand, as early as thirty years ago, it has been verified that VHb has terminal oxidase activity. After the *vgb* gene (*Vitreoscilla* hemoglobin gene) was transformed, under the condition of succinate or lactate for substrates, the *E. coli* mutant that lacks cytochrome *o* and *d* terminal oxidases could perform aerobic respiration and grow normally [16]. On the other hand, the activity of peroxidase was detected for VHb through in vitro experiments, and many factors (pH, temperature, etc.) can affect its activity [17,18]. In the following, in order to enhance the peroxidase activity of VHb for the application in biomedicine and dye decolorization, several mutated VHb that presenting high peroxidase activity under specific pH conditions were obtained [19]. Based on these mutants of VHb, it was found that the conserved amino acid residues 53 and 54 (glutamine and proline) in the distal pocket of VHb are closely related to its peroxide activity. Aiming for these two key catalytic sites, more and more designed mutants (P54R or Q53H/P54C) with higher peroxidase activity were obtained by site-directed mutations [20,21].

### 2.3. The Potential Sulfide Receptor and Storage

Different from the classic H_2_S-binding monomeric hemoglobin from *Lucina pectinate* [22], VHb exhibits unusual characteristics in its reactivity with H_2_S, such as steric constraints at position E11 (Leu), that play important roles in regulating the binding stability of H_2_S and VHb. The kinetic parameters for interaction between VHb and H_2_S were determined by UV-visible spectroscopic analysis and Resonance Raman (RR) spectroscopic analysis (*K_on_* = 1.2 × 10^5^ M^−1^S^−1^ and *K_off_* = 2.5 × 10^−4^ S^−1^), indicating that VHb serves as a potential sulfide receptor and has a storage function in the cells [23].

### 2.4. Other Functions

Besides the functions mentioned above, VHb also has the properties of lipid binding. VHb not only can interact with the monolayers formed by natural phospholipids but also reversibly binds to free fatty acids [24]. Since the binding site is located in the distal pocket of the heme, combination with lipids may affect the oxygen affinity of VHb and its physiological functions [24]. In addition, VHb can also interact with other intracellular enzymes or transcriptional regulators to increase their activities or activate the downstream metabolic pathways (Table 1).

## 3. Structure and Bioinformatics Analysis of VHb and Its Mutants

### 3.1. The Structure of VHb and Its Mutants

Based on the analysis of crystal structure, VHb forms six α-helix regions (A, B, E, F, G and H), which is different from other eukaryotic hemoglobins with eight α-helix regions (A, B, C, D, E, F, G and H), and has a unique distal heme pocket [28]. In addition, there are four residues (TyrB10, GlnE7, ProE8 and LeuE11) that are closely related to the oxygen-binding property [29]. Notably, unlike most eukaryotic hemoglobins, the distal His (E7) residue in VHb is substituted by Gln residue, which cannot form hydrogen bonds with oxygen [30]. Furthermore, the Gln (E7) residue in VHb is responsible for the disorder of the D-helix region that forms between the polypeptide segment from Phe-43 (CD1) to Leu-57 (E11), leading to the weaker affinity to oxygen, higher oxygen dissociation constant (*K_off_*) and rapid rate of oxygen transfer [30].

Apart from the wild-type VHb, the effect of key amino acid residues on the structure and function of VHb mutants was studied through site-directed mutagenesis. At first, the Tyr-29 (B10) played an important role in maintaining the stability of oxygen binding [31]. Next, the structure of the TyrB10Phe mutant is almost indistinguishable from the wild type, and the structure related to D-region ordering and E7 chain of the TyrB10Ala mutant is significantly different from the wild type [31]. Moreover, VHb also had a unique proximal heme pocket, with the structure being formed by a hydrogen-bonding network consisting of HisF8-TyrG5-GluH23 and TyrG5-TyrH12 [32]. In addition, the TyrG5Phe and TyrG5Leu mutants cannot form a stable oxygenated state and do not exhibit any nitric oxide dioxygenase activity [32]. However, the TyrH12Phe and TyrH12Leu mutants showed little effect on the oxygen-binding capacity, which is inconsistent with the previous predicted results that TyrH12Leu mutation could enhance oxygen diffusion and accumulation [32].

### 3.2. The Homology Analysis of VHb

It has been reported that VHb has a lower homology with eukaryotic hemoglobins and the highest homology only can reach 24% (leghemoglobin from *Lupinus luteus*). However, after the alignment of amino acid sequence between VHb and prokaryotic proteins, eight categories of bacterial homologous proteins were found, including bacitracin resistance protein BacA, hemoglobins (Hb), hypothetical protein (HP), NO-inducible flavohemoprotein (NOIFHP), flavohemoprotein (FHP), cytochrome *o* (Cyo), nitric oxide dioxygenase (NOD) and dihydropteridine reductase (DHPR).

**Figure 1:**
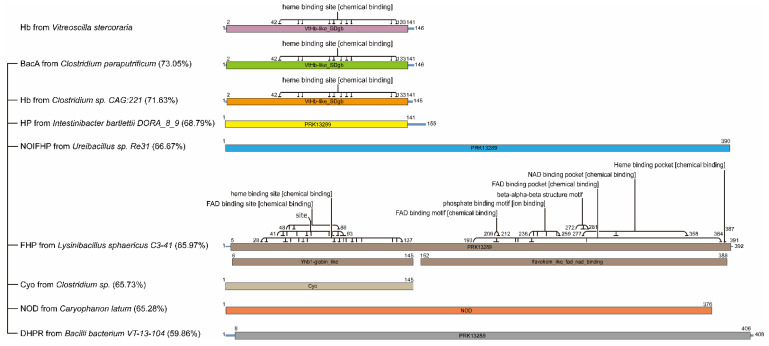
The homology analysis of VHb. The accession numbers in GenBank of eight homologous proteins and their homology with VHb: BacA from *C. paraputrificum* (WP_027099064.1, 73.05%), Hb from *Clostridium* sp. CAG:221 (CDB15533.1, 71.63%), HP from *I. bartlettii* DORA_8_9 (ETI93048.1, 68.79%), NOIFHP from *Ureibacillus* sp. Re31 (WP_191706693.1, 66.67%), FHP from *L. sphaericus* C3-41 (ACA41869.1, 65.97%), Cyo from *Clostridium* sp. (SCK00776.1, 65.73%), NOD from *C. latum* (WP_066464548.1, 65.28%), and DHPR from *B. bacterium* VT-13-104 (KKE77556.1, 59.86%). Hb: hemoglobin; BacA: bacitracin resistance protein; HP: hypothetical protein; NOIFHP: NO-inducible flavohemoprotein; FHP: flavohemoprotein; Cyo: cytochrome *o*; NOD: nitric oxide dioxygenase; DHPR: dihydropteridine reductase; VtHb: VHb, Vitreoscilla hemoglobin; SDgb: single-domain globin; PPK13289: belongs to the superfamily cl36224 and may span more than one domain. FAD: flavin adenine dinucleotide; NAD: nicotinamide adenine dinucleotide; Yhb1-globin_like: a globin domain such as the globin domain of the *Saccharomyces cerevisiae* flavohemoglobin (Yhb1p). Different colors represent different proteins or domains.

Among eight homologous categories, the proteins with highest homology with VHb were selected, including BacA from *Clostridium paraputrificum* (WP_027099064.1, 73.05%), Hb from *Clostridium* sp. CAG:221 (CDB15533.1, 71.63%), HP from *Intestinibacter bartlettii* DORA_8_9 (ETI93048.1, 68.79%), NOIFHP from *Ureibacillus* sp. Re31 (WP_191706693.1, 66.67%), FHP from *Lysinibacillus sphaericus* C3-41 (ACA41869.1, 65.97%), Cyo from *Clostridium* sp. (SCK00776.1, 65.73%), NOD from *Caryophanon latum* (WP_066464548.1, 65.28%), and DHPR from *Bacilli bacterium* VT-13-104 (KKE77556.1, 59.86%) (Figure 1).

Based on the analysis of conserved domains, the results show that BacA from *C. paraputrificum*, Hb from *Clostridium* sp. CAG:221, and FHP from *L. sphaericus* C3-41 all contain heme binding sites. Particularly, there are NAD (nicotinamide adenine dinucleotide) and FAD (flavin adenine dinucleotide) binding sites on FHP from *L. sphaericus* C3-41, which are very similar to the structure of VHb. BacA from *C. paraputrificum* and Hb from *Clostridium* sp. CAG:221 belong to the VtHb-like_SDgb (VHb-like_SDgb) protein family. BacA has the activity of undecaprenyl pyrophosphate phosphatase that is involved in the cell wall synthesis [33]. In addition, BacA can interact with lipids, which is similar to the function of VHb [24]. HP from *I. bartlettii* DORA_8_9, NOIFHP from *Ureibacillus* sp. Re31, FHP from *L. sphaericus* C3-41, and DHPR from *B. bacterium* VT-13-104 belong to PPK13289, a member of the cl36224 protein superfamily and may span more than one domain. Although no conserved domain was obtained by alignment, the function of NO-dioxygenase (NOD) from *C. latum* and Cyo from *Clostridium* sp. is similar with VHb [16,25], indicating some potential functional domains still remain to be discovered.

## 4. The Heterologous Expression of VHb

### 4.1. The Regulation of VHb Expression by Its Native Promoter

After the *vgb* gene encoding VHb was identified in *Vitreoscilla* [2], VHb was first heterologously expressed by its native promoter in *E. coli* [3]. It is worth noting that the expression of VHb was induced under hypoxic conditions both in *Vitreoscilla* and *E. coli*, indicating the oxygen-sensitive regulatory mechanism for its native promoter. In the following, it is found that there are binding sites of the oxygen-responsive transcriptional regulators OxyR, Fnr, ArcA, and Crp on the *vgb* promoter. Under oxygen-limited conditions, Fnr, ArcA, and Crp activate the expression of the *vgb* gene independently or in combination to promote oxygen supply and enhance respiratory activity [34]. Under high aeration, OxyR can not only down-regulate the transcription of the *vgb* gene by binding to the *vgb* promoter, but also interacts with VHb to convert it into an oxidized state that can positively regulate the expression of genes involved in oxidative stress and enhance the ability of cells to resist oxidative stress [26].

### 4.2. The Strategies to Improve VHb Expression

Due to the promising effect of oxygen delivery on the growth of strains and the synthesis of useful products, the expression levels of VHb should be adjusted for different kinds of microbial hosts [35]. At present, three factors that can significantly influence VHb expression have been optimized, including the copy number of the *vgb* gene, the vector copy number, and the promoter strength (Table 2).

At first, based on the effect of different VHb expression levels on the growth of *E. coli*, the suitable copy number of the *vgb* gene was determined. The result showed that the increased integrated copies of the *vgb* gene under the control of the *vgb* promoter cannot improve cell growth [36]. Therefore, the single copy of *vgb* gene was generally adopted in the following metabolic engineered strains. Next, three different recombinant *E. coli* strains (harboring low, middle, and high copy numbers of vectors containing the *vgb* gene, respectively) were constructed to improve the titer of ethanol. The results showed that the titer of ethanol was inversely proportional to the expression level of VHb and the highest titer of ethanol was obtained by the lowest VHb co-expression [35].

At last, the efficient expression of the *vgb* gene was achieved by selecting appropriate promoters. The native *vgb* promoter works in several Gram-negative bacteria, including eight-tandem *vgb* promoter P8vgb in *E. coli*, *Halomonas bluephagenesis* and *Halomonas campaniensis* [37,38]. The specific promoters that have been chosen for other bacteria include *trc* promoter in *E. coli* [39,40], *tac* promoter in *E. coli* and *Thialkalivibrio versutus* [41,42,43], P_phaC1-j5_ promoter in *Cupriavidus necator* [44], and P43 promoter in *Bacillus subtilis* [45]. Fungal promoters that have been used for expression in fungi include: tubulin promoter in *Aurantiochytrium* sp. [46], constitutive *ermE* promoter in *Streptomyces* sp. [47], and *AOX1* promoter in *Pichia pastoris* [48,49]. In addition, the CaMV35S promoter has been chosen in higher plant systems [9,50].

## 5. The Effect of VHb Expression on Cell Metabolism

The result of transcriptomics showed that the expression of VHb can affect hundreds of genes in *E. coli*, especially for the genes involved in central carbon and energy metabolism [41]. In addition, under the conditions of limited oxygen and glucose as the sole carbon in *E. coli*, the analysis of metabolic flux distribution further demonstrated that the expression of VHb leads to dominant carbon flux in the pentose phosphate pathway (PPP), while the remaining carbon flux is guided toward the tricarboxylic acid (TCA) cycle [51]. Further research showed that the TCA cycle in *vgb*^+^ cells of *E. coli* can function in a branched manner under hypoxic conditions [52]. Along with the increasing carbon flux in PPP, more NADPH was produced and a net NADH flux is generated by the NADH/NADPH transhydrogenase in *vgb*^+^ cells under microaerobic conditions [51]. Moreover, VHb delivers oxygen to the respiratory chain, the respiratory activity was enhanced, the ratio of NAD^+^/NADH and ATP generation was improved [7,53]. Furthermore, by-products in the fermentation process were significantly reduced (acetate ~25%, ethanol ~49%, formate ~68%, lactate ~72%, and succinate ~50%) and growth yield increased 35% in *vgb*^+^ cells [51]. Especially for acetate, the following transcriptional analysis showed that the transcriptional levels of the glyoxylate shunt genes were also decreased [54].

## 6. Applications of VHb in Biotechnology

**Figure 2:**
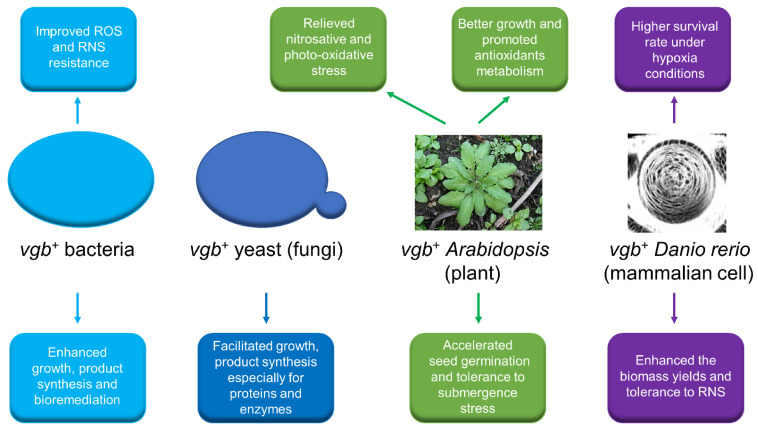
The potential applications of VHb in the field of metabolic engineering for bacteria [55,56], fungi [48,49], plants [57] and animals [58]. ROS: reactive oxygen species; RNS: reactive nitrogen species.

Dependent on the robust capacity of oxygen transport under hypoxic conditions, VHb has been widely used for the improvement of biosynthesis, cell growth and bioremediation (Figure 2).

### 6.1. VHb in Biotechnological Productions

VHb has been used for some important value-added products such as acetoin, butanediol and L-asparaginase under hypoxic conditions. For acetoin and butanediol, the *vgb*^+^ engineered *Enterobacter aerogenes* showed an enhancement of 83% in accumulation of acetoin and butanediol compared to the control strain without *vgb* [59]. In addition, an increase of 70% on the production of L-asparaginase was achieved in *Pseudomonas aeruginosa* by introducing the *vgb* gene [60]. Furthermore, many processes of ethanol production from pure sugars and industrial waste (corn, molasses, whey, whey powder, etc.) can be enhanced by VHb expression [61]. By combining immobilization with VHb, the immobilized engineered *E. coli* increased by 47% in the medium with an intermediate concentration of lactose from whey powder [62]. After the optimization of immobilization conditions (10% bead inoculation) and the medium composition (8% lactose from whey powder), the *vgb*^+^ immobilized *E. coli* displayed a higher titer of ethanol to 4.64% [63].

Besides the above-mentioned products, the production of other target products, including antibiotics, enzymes, organic acids and polysaccharides, can also be significantly increased through heterologous expression of VHb (Table 3). For antibiotics, the synthesis of pyocyanin and rifamycin B can be increased 3-fold in *E. coli* [64] and 2.2-fold in *Amycolatopsis mediterranei* [65], respectively. For enzymes, the expression of lipase 2, coenzyme Q10 and xylanase can be enhanced by 87.84% in *P. pastoris* [48], 71% in *Rhodobacter sphaeroides* [66] and 31% in *P. pastoris* [49], respectively. For organic acids, the VHb expression has a greater effect on arachidonic acid (8-fold) in *Mortierella alpina* [67], docosahexaenoic acid (2.74-fold) in *Aurantiochytrium* sp. [68], and ganoderic acid (1.4-fold) in *G. lucidum* [69]. For polysaccharides, several complex compounds, including bacterial cellulose (58.6%) in *Gluconacetobacter xylinus* [70], pullulan (42.08%) in *Aureobasidium melanogenum* [71], β-glucan (12.9–24.0%) in *Lentinula edodes* [72], and 6-(N-hydroxyethyl)-amino-6-deoxy-alpha-l-sorbofuranose (11.89%) in *Gluconobacter oxydans* [10] can be efficiently obtained, respectively.

### 6.2. VHb in Plants

The expression of VHb was also used to improve the waterlogging tolerance of higher plants. Under waterlogging conditions, VHb expressed in *Zea mays* L. seedlings can induce a higher activity of peroxidase and alcohol dehydrogenase 1 that are correlation with tolerance to oxidative stress and enhance the growth performance of plants (seedling height, root dry weight, primary root length, etc.) [9]. In addition, the expression of VHb can regulate the transcription of endogenous genes that refer to antioxidant biosynthesis and oxygen metabolism in plant cells, and protect cells from oxidative damage [57]. Furthermore, the biosynthesis of ascorbate and the tolerance to photo-oxidative stress were enhanced in VHb-expressing *Arabidopsis* cells [57]. VHb also plays a positive role in some other higher plants, including the enhancement of productivity and resistance to the herbicide glyphosate in *Oryza sativa* L. [78], and the improvement of seed germination and tolerance to submergence stress in cabbage (*Brassica oleracea var. capitata* L.) [79]. However, the expression of VHb does not always have a positive effect on plants. In the case of *Hordeum vulgare*, it exhibited a slower germination rate and impaired rooting of seedlings, which may be attributed to the removal of a significant signaling molecule (NO) related to seed germination and root formation by VHb [80]. In addition, the introduction of the *vgb* gene into *Populus alba* L. did not improve tolerance to submergence, oxidative and nitrosative stresses [81]. Therefore, it is necessary to carefully investigate the influence of the genetic manipulation of oxygen metabolism of higher plants on the physiological and biochemical characteristics of cells in order to evaluate the true value of VHb application for higher plants.

### 6.3. VHb in Mammalian Cells

There are also several successful reports on the application of VHb engineering in animal cells, including increased tissue plasminogen activator (~40–100%) in Chinese hamster ovary cells [82], increased survival rate (*vgb*^+^-34.57~92% and *vgb*^−^-15.69~65%) in *Danio rerio* [58], and increased biomass yields by 60% and lactate decrease of 40% in Chinese hamster ovary cells [7]. Since the metabolism of animals is much more complicated than that of microorganisms, the application of VHb in the field of animals has been relatively slow in recent years.

### 6.4. VHb in Biodegradation Applications

VHb expression was frequently applied in biodesulfurization, degradation of pesticides, and wastewater treatment (Table 4). Dependent on the function of sulfide receptor and storage [23], the expression of VHb in *Rhodococcus erythropolis* (desulfurization bacterium) presented a higher desulfurization ratio than the control (*vgb*^+^-37.5% and *vgb*^−^-20.5%) under hypoxic conditions [83]. In the following, an increase of 11.7 ± 1.8% on the rate of thiosulfate scavenge was achieved in *T. versutus* by introducing the *vgb* gene [42]. In addition, the co-culture of desulfurization bacteria is also an effective sulfur degradation method [84]. For example, co-culture of *Paenibacillus* strains (*vgb*^+^) showed a stronger growth than the control (*vgb*^−^) under the conditions of dibenzothiophene [11]. Moreover, introduction of the *vgb* gene into *Pseudomonas putida* can improve its pesticide degradation function under oxygen-limited conditions, including simultaneous degradation of methyl γ-hexachlorocyclohexane and parathion [85], the removal of 1,2,3-trichloropropane [86], and the simultaneous elimination of carbamates, pyrethroids, and organophosphates [87]. As for wastewater treatment, the *vgb*^+^ engineered *Burkholderia cepacia* strain was first applied with a parallel membrane bioreactors system and displayed a significant increase in the degradation efficiency of 2-chlorobenzoic acid (*vgb*^+^ ~94–97% and *vgb*^−^ ~67–85%) [88]. In addition, based on the activity of peroxidase, one of the VHb variants (Q53H/P54C) shows excellent prospects for treating wastewater contaminated by textile dyes [21]. Furthermore, the *b*-type heme derived from VHb is quite beneficial for the activity of hemoglobin in activated sludge, which functions as oxidase or peroxidase and plays an important role in traditional aerobic wastewater treatment [89].

## 7. Conclusions and Future Perspectives

VHb is a special bacterial hemoglobin that can interact with terminal oxidase to provide enough oxygen for cell growth. Based on analyses of its properties and crystal structures, VHb has been applied in the field of metabolic engineering for microorganisms, plants, and animals to achieve high-cell-density fermentation and to enhance product synthesis and stress tolerance under oxygen-limited conditions. By the optimization of its expression strategies, the effect of VHb was further improved, allowing VHb technology to be used for more and more products.

In the future, there are four possible directions for the development of VHb application. Firstly, the precursors of heme (5-aminolevulinic acid) could be supplemented or the biosynthesis of heme could be enhanced to increase the activity of VHb because many microorganisms cannot supply enough heme for VHb expression. In the case of eukaryotic hemoglobins, the active *Arenicola Marina* globin chains were efficiently expressed by the addition of 5-aminolevulinic acid in *E. coli* [92]. In addition, an improvement of human hemoglobin production was obtained in *S. cerevisiae* with an enhanced heme synthesis pathway [93]. Secondly, the addition of iron and transport of iron over cell membranes also have a positive effect on hemoglobin production. The hemoglobin of β-thalassemic mice was increased with the exogenous addition of iron [94]. Thirdly, more and more VHb mutants with improved characteristics can be selected by protein engineering and high throughput screening. Furthermore, the expression of VHb will also contribute extra metabolic burden, but the optimization of promoter, substrate and inducer can significantly relieve this adverse effect on the host [95,96]. Finally, more research on the regulatory mechanism of VHb on oxygen-response is needed to expand its application in other areas.

## Figures and Tables

**Table 1:** Interactions between VHb and intracellular enzymes or regulators.

Enzymes/Regulators	Functions	References
Flavoreductase	Relieve nitrosative stress	[25]
Transcriptional regulators (OxyR, Fnr, ArcA, Crp)	Transcriptional regulation	[26]
2,4-dinitrotoluene dioxygenase	Enhance dioxygenase activity	[27]

OxyR: oxidative stress regulator; Fnr: fumarate and nitrate reductase; ArcA: aerobic respiration control A; Crp: catabolic repressor protein.

**Table 2:** The strategies of VHb expression.

Strain	Expression Strategies	References
*Escherichia coli*	Free; inducible; *vgb* promoter	[36]
*E. coli*	Free; inducible; *vgb* promoter	[35]
*E. coli*	Free; inducible; P8vgb	[37]
*E. coli*, *Halomonas bluephagenesis* and *Halomonas campaniensis*	Free; inducible; P8vgb	[38]
*E. coli*	Integrative; constitutive; *trc* promoter	[39]
*E. coli*	Integrative; inducible; *trc* promoter	[40]
*E. coli*	Free; inducible; *tac* promoter	[41]
*Thialkalivibrio versutus*	Free; constitutive; *tac* promoter	[42]
*E. coli*	Free; constitutive; *tac* promoter	[43]
*Cupriavidus necator*	Free; constitutive; P_phaC1-j5_ promoter	[44]
*Bacillus subtilis*	Free; constitutive; P43 promoter	[45]
*Aurantiochytrium* sp.	Integrative; constitutive; tubulin promoter	[46]
*Streptomyces* sp.	Integrative; constitutive; *ermE* promoter	[47]
*Pichia pastoris*	Integrative; inducible; *AOX1* promoter	[48]
*P. pastoris*	Integrative; inducible; *AOX1* promoter	[49]
*Arabidopsis* and *Zea mays* L.	Integrative; constitutive; CaMV35S promoter	[9]
*Hyoscyamus niger*	Integrative; constitutive; CaMV35S promoter	[50]

Free: intracellular free expression by plasmid; Integrative: intracellular integrative expression by chromosomally integration; Inducible: intracellular inducible expression by the addition of inducers; Constitutive: intracellular constitutive expression that do not need inducers. P8vgb: eight-tandem *vgb* promoter; *trc* promoter: *trp and lac UV5* promoter hybridized; *tac* promoter: a hybrid between the *trp* and *lac* promoters; P_phaC1-j5_ promoter: a hybrid between P_phaC1_ and P_j5_ promoter; tubulin promoter: a promoter amplified from the genome of *Aurantiochytrium* sp.; *ermE* promoter: a strong constitutive promoter commonly used in *Streptomyces* sp.; *AOX1* promoter: methanol-inducible promoter commonly used in *P. pastoris*; CaMV35S promoter: the 35S promoter from the plant pathogen cauliflower mosaic virus.

**Table 3:** The titer of products increased by the expression of VHb.

Products		Enhancement	Strain	References
Alcohols	Ethanol	~362%	*E. coli*	[73]
		~118%	*E. coli*	[35]
		~60%	*E. coli*	[74]
		~47%	*E. coli*	[62]
		~(41-83%)	*E. coli*	[63]
	Butanediol	~83%	*Enterobacter aerogenes*	[59]
	Erythritol	~26.13%	*Yarrowia lipolytica*	[75]
Antibiotics	Pyocyanin	~3-fold	*E. coli*	[64]
	Rifamycin B	~2.2-fold	*Amycolatopsis mediterranei*	[65]
Enzymes	Lipase 2	~87.84%	*P. pastoris*	[48]
	Coenzyme Q10	~71%	*Rhodobacter sphaeroides*	[66]
	Xylanase	~31%	*P. pastoris*	[49]
	L-asparaginase	~70%	*Pseudomonas aeruginosa*	[60]
Acids	Arachidonic acid	~8-fold	*Mortierella alpina*	[67]
	Docosahexaenoic acid	~2.74-fold	*Aurantiochytrium* sp.	[68]
	Ganoderic acid	~1.4-fold	*Ganoderma lucidum*	[69]
	S-adenosylmethionine	~67%	*S. cerevisiae*	[76]
	Glucaric acid	~28.76%	*S. cerevisiae*	[77]
	L-phenylalanine	~16.6%	*E. coli*	[43]
Polysaccharides	Bacterial cellulose	~58.6%	*Gluconacetobacter xylinus*	[70]
	Pullulan	~42.08%	*Aureobasidium melanogenum*	[71]
	β-glucan	~(12.9–24.0%)	*Lentinula edodes*	[72]
	6-(N-hydroxyethyl)-amino-6-deoxy-alpha-l-sorbofuranose	~11.89%	*Gluconobacter oxydans*	[10]
Others	Polyhydroxybutyrate	~71.5%	*C. necator*	[44]
	Acetoin	~83%	*Enterobacter aerogenes*	[59]

Enhancement: the ratio of the increase in the product titer of *vgb*^+^ strain relative to the control (% or fold).

**Table 4:** The applications of VHb in biodegradation.

Compounds	Strain	References
Dibenzothiophene	*Rhodococcus erythropolis*	[83]
Thiosulfate	*T. versutus*	[42]
Dibenzothiophene	*Paenibacillus*	[11]
Pesticides	*Pseudomonas putida*	[85,86,87]
2-chlorobenzoic acid	*Burkholderia cepacia*	[88]
Benzene, toluene and xylene	*Pseudomonas aeruginosa*	[90]
Cadmium	*Enterobacter aerogenes*	[91]
